# Petrophysical examination of CO_2_-brine-rock interactions—results of the first stage of long-term experiments in the potential Zaosie Anticline reservoir (central Poland) for CO_2_ storage

**DOI:** 10.1007/s10661-014-4215-6

**Published:** 2014-12-18

**Authors:** Radosław Tarkowski, Magdalena Wdowin, Maciej Manecki

**Affiliations:** 1 The Mineral and Energy Economy Research Institute of The Polish Academy of Sciences, Wybickiego 7, 31-261 Kraków, Poland; 2 AGH-University of Science and Technology, Mickiewicza 30, 30-059 Kraków, Poland

**Keywords:** CO_2_ storage, Reservoir sandstones, Sealing rocks

## Abstract

The objective of the study was determination of experiment-induced alterations and changes in the properties of reservoir rocks and sealing rocks sampled from potential reservoir for CO_2_. In the experiment, rocks submerged in brine in specially constructed reactors were subjected to CO_2_ pressure of 6 MPa for 20 months at room temperature. Samples of Lower Jurassic reservoir rocks and sealing rocks (sandstones, claystones, and mudstones) from the Zaosie Anticline (central Poland) were analysed for their petrophysical properties (specific surface area, porosity, pore size and distribution) before and after the experiment. Comparison of the ionic composition the brines before and after the experiment demonstrated an increase in total dissolved solids as well as the concentration of sulphates and calcium ions. This indicates partial dissolution of the rock matrix and the cements. As a result of the reaction, the properties of reservoir rocks did not changed significantly and should not affect the process of CO_2_ storage. In the case of the sealing rocks, however, the porosity, the framework density, as well as the average capillary and threshold diameter increased. Also, the pore distribution in the pore space changed in favour of larger pores. The reasons for these changes could not be explained by petrographic characteristics and should be thoroughly investigated.

## Introduction

In recent times, opinions regarding the usage of fossil fuels have been changing in many countries. This has been expressed, for example, in obligations adopted to reduce greenhouse gas emissions and the ratification of the Kyoto Protocol by many governments (Nimtz et al. 2010). Carbon capture and storage (CCS) is a key strategy within the portfolio of actions aimed at reducing CO_2_ emissions to the atmosphere. Geological storage in the deep saline aquifer is one of the most promising options on a regional to global scale (Fischer et al. 2010; IPCC 2005).

The phenomena of CO_2_ injection effects on reservoir fluids and rocks are related to many aspects such as the kinetics of CO_2_ dissolution in reservoir fluids, the mechanisms and kinetics of mineral trapping (Kampman et al. 2014), the appearance of new mineral phases (Fischer et al. 2010; Wdowin et al. 2014a), migration of gas within the reservoir (Riding and Rochelle 2009), evaluations of geological storage capacities (Aydin et al. 2010), the sealing properties of cap rocks, even impurities in injected gas (Pearce et al. 2014) and many others. The study of these processes is vital to planning safe underground CO_2_ storage operations. During preliminary studies on the usefulness of rocks for the geological storage of CO_2_, among the many problems to consider is the effect of the carbon dioxide on rocks and reservoir fluids (Tarkowski and Wdowin 2011; Wdowin et al. 2014b). The long-term interaction of injected CO_2_ in the deep reservoir is crucially dependent on the performance of the reservoir, cap, and sealing rock in light of the chemical reactions with CO_2_-charged brine. It is expected that supercritical CO_2_ will dissolve into the brine flowing through the reservoirs. The CO_2_-charged brine then becomes acidic and will react with the rocks, which would bring uncertainty to large-scale CO_2_ storage applications. Furthermore, CO_2_-brine-rocks interactions may potentially lead to changes in porosity and permeability, via chemically coupled mechanical effects (Hangx et al. 2013). Also important is estimation of the potential injection rates into a deep saline aquifer, taking into account the temporal variability of a CO_2_ stream produced from the operation of a power plant (Wiese et al. 2010).

Most of the previous studies on CO_2_-water-rock interaction have focused only on the reservoir rocks, primarily sandstone or carbonate reservoirs, that store the CO_2_ (Rochelle et al. 2004; Czernichowski-Lauriol et al. 1996a; Izgec et al. 2008). But geochemical reactions of the cap as well as sealing rock associated with the CO_2_ sequestration have been much less thoroughly studied.

So far, the effect of CO_2_ injection on physical rock properties is not well established. One of the numerous problems related to CO_2_ storage in various geological structures is its impact on rocks and reservoir fluids (Enick and Lara 1990; Czernichowski-Lauriol et al. 1996b; Fischer et al. 2013). When injected, CO_2_ interacts with the rocks and reservoir fluids; it is dissolved in reservoir liquids, reacts with the minerals of the rock matrix (Gunter et al. 1993), undergoes hydrodynamic trapping (Bertier et al. 2006), and if the sealing is not perfect, can leak into overlying lithologies (Gaus et al. 2005) or even to the surface (Wdowin et al. 2014a). Fluid-rock interaction processes may result in changes in porosity geometry and distribution, effective permeability, and capillary entry conditions—all of which influence and change the petrophysical and fluid transport properties (Zemke et al. 2010). Therefore, understanding of the CO_2_-brine-cap rock interactions is critical to the site selection, risk assessment, and the ultimate public acceptance of CO_2_ storage programs (Liu et al. 2012). The purpose of this study is to evaluate CO_2_-brine-rock (reservoir and sealing rocks) interactions resulting from long-term experiments by analysis of petrophysical properties from the point of view of their usefulness for CO_2_ storage.

## Materials and methods

An experiment on the interactions between rock, brine, and carbon dioxide was performed on a number of rock samples (sandstones, claystones, and mudstones) in custom-designed autoclaves. The grain framework morphology and petrophysical parameters of the rocks were determined before and after the experiment in order to identify the impact of the experiment on their reservoir and sealing properties.

Porosimetry analyses have allowed for a determination of the distribution of pores within the pore space preserved in the rocks and assessment of their hydraulic and reservoir properties. These parameters are crucial in appraising the suitability of the rocks for CO_2_ storage. For sealing rocks, it is essential that the porosity and the proportion of pores are not increasing, while in the case of reservoir rocks, it is desirable that the porosity and pore volume are not being reduced.

### Research material

**Fig. 1:**
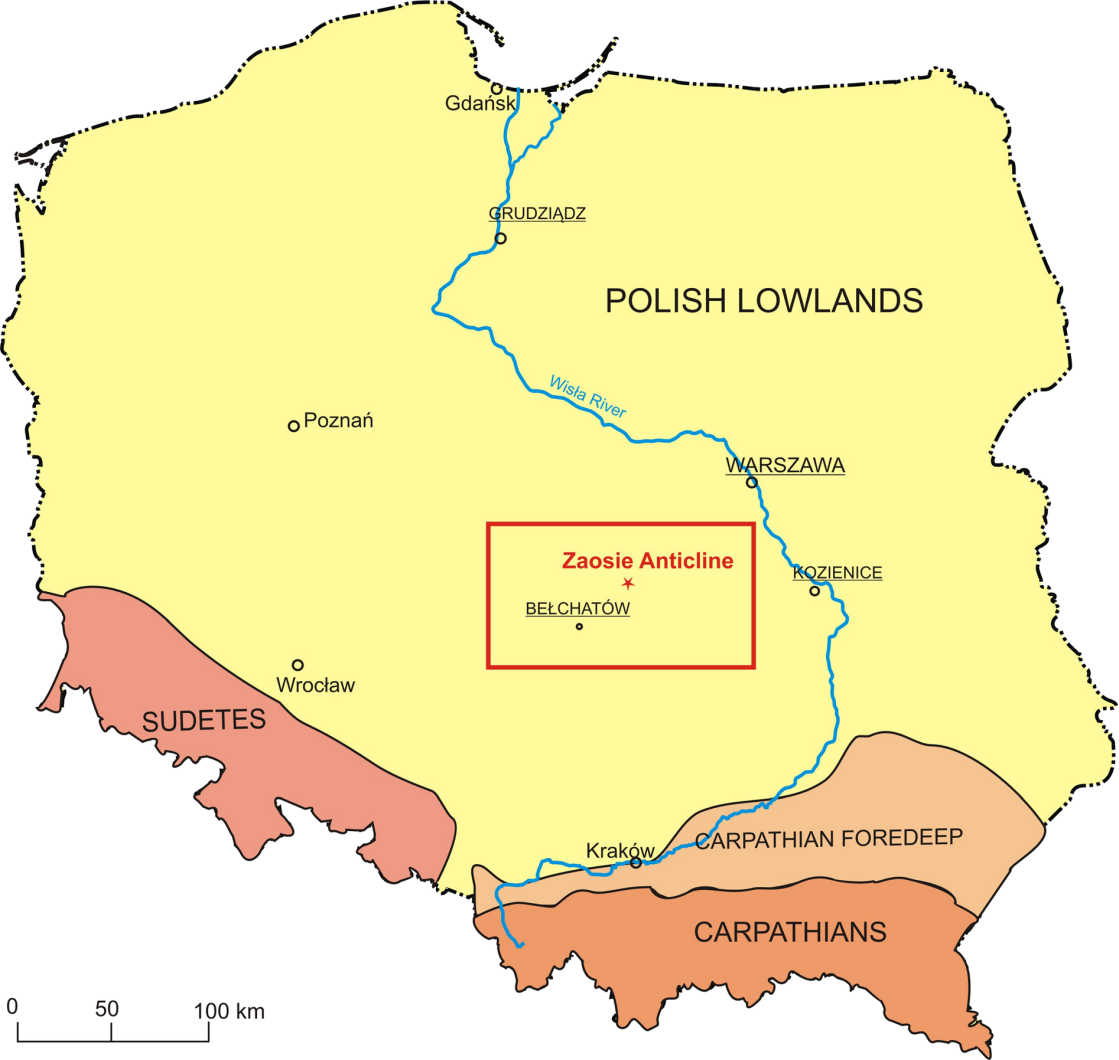
Location map of Zaosie Anticline as a potential site for CO_2_ storage

The tested samples represent Lower Jurassic rocks of potential reservoir (sandstones) and sealing (claystones and mudstones) series. They come from the Zaosie 2 and Buków 2 boreholes drilled within the Zaosie Anticline (Table 1). This is located in central Poland on the southwestern end of the Kuyavian Swell (Fig. 1), about 5–6 km north of Tomaszów Mazowiecki (Tarkowski ed. 2010). The Zaosie Anticline was chosen as one of the three potential sites for CO_2_ storage for the Bełchatów Power Plant. It was selected by six other European projects for the program implemented under the European Economic Plan for Recovery (Tarkowski et al. 2011; Labus et al. 2010). Due to the limited availability of research material and relatively homogeneous composition of the rock formations, four representative samples of drillcores were selected for the study: two from reservoir rocks and two from sealing formations.

**Table 1:** Reservoir and sealing rocks selected for the study

No.	Well	Depth [m]	Sort of sample	Age
1	Zaosie 2	838.10	Sandstone	Lower Jurassic (Upper Pliensbachian–Upper Sinemurian
2	Zaosie 2	912.40	Claystone	
3	Buków 2	1,435.60	Sandstone	Lower Jurassic (Lower Pliensbachian–Upper Sinemurian)
4	Buków 2	1,436.00	Mudstone

### The experiment

The experiment was conducted in a specially designed test apparatus. The samples of rocks were placed on Petri dishes in glass cylinders filled with brine and inserted into four, custom-built, stainless steel, high-pressure autoclaves (1.8 dm^3^ each). The solution was not in direct contact with the walls of the autoclaves. Experiments with samples of reservoir sandstones and sealing rocks (claystones or mudstones) from both the Zaosie 2 and Buków 2 boreholes were run in separate autoclaves. The chemical composition of the brine corresponds to the brines from Jurassic rocks of this region (based on PGI-NRI data). The contents of individual ions were as follows: Cl^−^—741 mg/dm^3^, SO_4_^2−^—396 mg/dm^3^, HCO_3_^−^—195 mg/dm^3^, CO_3_^2−^—334 mg/dm^3^, Ca^2+^—1.2 mg/dm^3^, Na^+^—807 mg/dm^3^, K^+^—110 mg/dm^3^. Tightly closed reactors were connected with a CO_2_ tank at a pressure of 6 MPa. The experiment lasted for 20 months at room temperature.

### Methods

To monitor the alterations within the grain framework of the studied rocks, an FEI Quanta 200 FEG scanning electron microscope was used, equipped with a system for EDS EDAX microprobe analysis, as well as with a backscattered electron detector (YAG BSE). Porosimetry analysis was performed in an AUTO PORE 9220 mercury porosimeter (Micromeritics). The principle of capillary pressure measurements consisted of injecting non-wetting liquid (mercury) into the rock samples under clearly defined pressures, according to a predetermined pressure table. Based on the measurements in the mercury porosimeter, a cumulative curve was obtained which shows the relationship between the applied pressure and the volume of injected mercury. The maximum pressure applied was 30,000 psi which allowed for the injection of mercury into the micropores of a minimum diameter of 0.009 μm (9 nm). The volume of mercury injected under increasing pressure into smaller and smaller pores in the pore space of the rock was determined during the measurements. The sizes (diameters) of pores into which mercury was injected were calculated using the Washborne equation:

$$ D = \left(1/p\right)\ y\  \cos \phi $$

where

*D*: Diameter of the pores
*P*: Pressure used (according to pressure tables)
*ϕ*: Mercury-rock contact angle
*y*: Surface tension.

Based on the analysis of cumulative curves (i.e. capillary pressure curves), a number of parameters characterising the pore space structure of the rock were calculated including dynamic porosity, specific surface area, average capillary size, and threshold diameter (threshold pressure). Besides using Hg porosimetry, a proportion of pores greater than 1 μm as well as framework density and bulk density were determined. Additionally, analysis of pore space distribution was carried out. Petrophysical analyses were performed on raw rock samples and on samples used for the experiment.

The roughness of the rock surface of the samples has a certain effect on the measured dynamic porosity and the amount of some other petrophysical parameters of the rocks. In order to eliminate the slight boundary effect in the porosimetry analysis, the results of the measurements were recalculated by subtracting the part of mercury that filled the cavities on the surface (treated by the Auto Pore software as real pores existing in the rock structure) from the total volume of mercury that was injected into the pore space.

Analysis of the brine was performed before and after the experiment to determine the changes in the proportions of individual ions, which is related to the reactions including dissolution or precipitation of various mineral phases. Determinations of individual ions were performed based on the following national and European standards: chlorides—PN-ISO 9297:1994, sulphates—PN-ISO 9280:2002, bicarbonates—PN-EN ISO 9963:2001 + Ap-1:2004, calcium—PN-ISO 6058:1999, magnesium—PN-ISO 6059:1999, sodium—PN-EN ISO 11885:2009, and potassium—PN-EN ISO 11885:2009. Mineralisation (total dissolved solids, TDS) was determined based on the standards PBE-12 Ed. VI, dated June 28, 2007.

## Research results

### SEM-EDS observations

**Fig. 2:**
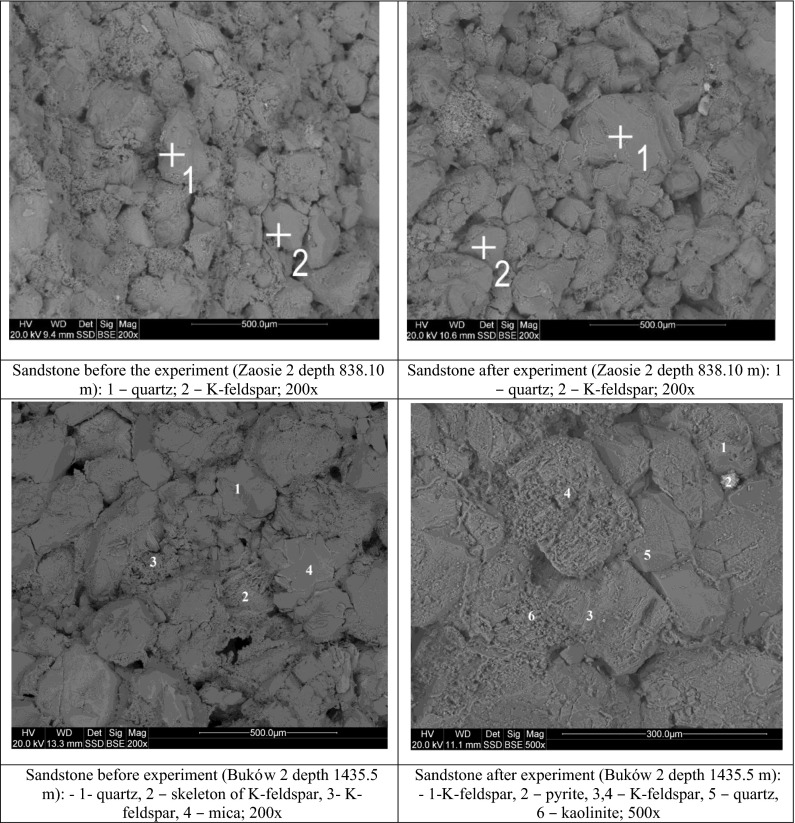
Representative SEM photomicrographs of reservoir rocks before and after the experiment

Analysed reservoir rocks (Fig. 2) (Zaosie 2 depth 838.10 m and Buków 2 depth 1,435.50 m) are representative of moderately and well-sorted quartz sandstones. The grain framework typically contains quartz, K-feldspars, and accessory micas. The cement is composed of kaolinite with minor admixture of carbonate minerals. Locally, a matrix is observed. Feldspar grains and their weathered skeletons are often kaolinitised. This is observed in sandstones both before and after the experiments. The most pronounced alteration resulting from the experiment is slight reduction in the amount of cement. Additionally, precipitation of fine-grain aluminosilicates (kaolinite) is apparent in SEM images.

**Fig. 3:**
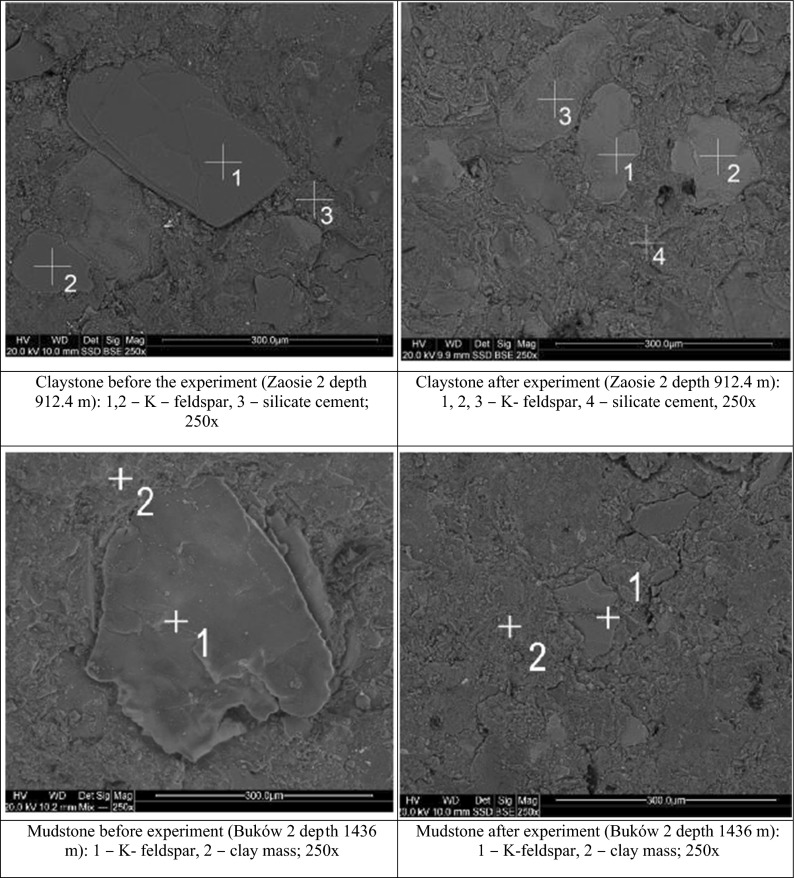
Representative SEM photomicrographs of sealing rocks before and after the experiment

The sealing rocks are representative of claystones and mudstones (Fig. 3). Their grain framework contains quartz, feldspars, micas, chlorites, and cement composed of silicate and clay minerals. No alterations resulting from the experiment were observed with the use of SEM in these claystones and mudstones.

Besides, in reservoir and sealing rocks, small amounts of halite precipitated from brine are observed.

### Petrophysical analyses

#### Reservoir rocks

**Fig. 4:**
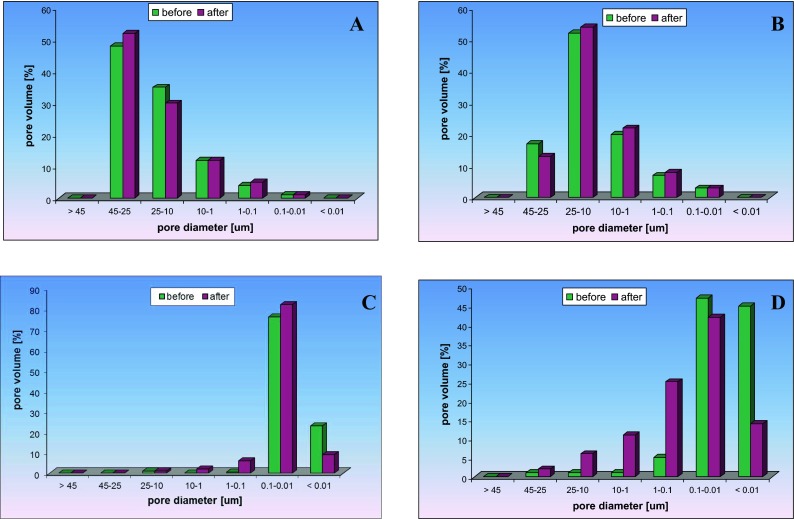
Distribution of pore space before and after experiment: **a** sandstone from Zaosie 2, depth 838.10 m; **b** sandstone from Buków 2, depth 1,435.5 m; **c** claystone from Zaosie 2, depth 912.40 m; **d** mudstone from Buków 2, depth 1,436 m

Petrophysical analyses (Table 2) showed that the sandstones (before the experiment) are characterised by low specific surface area of 0.26–0.42 m^2^/g and relatively high porosity exceeding 20 %. Rock samples from the Zaosie 2 borehole show porosity of about 24.5 %, and the rocks from Buków 2 borehole 21.4 %. These rocks also have similar threshold diameters of 30 μm and framework densities of 2.64 g/cm^3^. Sandstones from the Zaosie 2 borehole have slightly lower bulk density of 1.96 g/cm^3^ compared to those from the Buków 2 borehole at 2.05 g/cm^3^. The sandstones are characterised by variability in the pore space above 1 μm (Fig. 4). The predominant pore diameters for the sandstones from the Zaosie 2 borehole are 25–45 and 10–25 μm (total of 83 %) while the dominant pore diameters for the sandstones from Buków 2 are between 1 and 45 μm (total of 89 %).

**Table 2:** Petrophysical parameters determined by Hg porosimetry method, with boundary effect accounted for

No.	Well name	Examination stage	Depth [m]	Lithology	Surface area [m^2^/g]	Porosity Hg porsimeter [%]	Pores >1 μm [%]	Average capillary [μm]	Treshold diameter [μm]	Framework density [g/cm^3^]	Bulk density [g/cm^3^]
1	Zaosie 2	Before	838.10	Sandstone	0.26	24.5	95	2.00	30	2.650	1.963
2	Zaosie 2	After	838.10		0.22	24.0	94	2.39	30	2.626	1.964
3	Zaosie 2	Before	912.4	Claystone	5.03	5.0	1	0.017	0.05	2.569	2.436
4	Zaosie 2	After	912.4		4.87	7.6	3	0.026	0.07	2.620	2.416
5	Buków 2	Before	1,435.50	Sandstone	0.42	21.4	89	1.026	32	2.644	2.058
6	Buków 2	After	1,435.50		0.44	17.6	89	0.787	30	2.457	2.023
7	Buków 2	Before	1,436.0	Mudstone	3.95	3.1	3	0.014	0.05	2.536	2.452
8	Buków 2	After	1,436.0		4.87	9.1	19	0.033	0.35	2.594	2.349

The alterations resulting from the experiment only slightly influenced some of the petrophysical parameters of the reservoir rocks (Table 2). The porosity and the framework density decreased only in the sandstones from Buków 2—from 21.4 to 17.6 % and from 2.64 to 2.46 g/cm^3^. Moreover, in the case of samples from the Buków 2 borehole, a decrease in the values of average capillary and threshold diameter was also observed. Each sandstone revealed minimal changes in the pore size distribution (Fig. 4). In the sandstone from Zaosie 2, the proportion of pores in the range of 25–45 μm increased (from 48 to 52 %), while the proportion of pores in the range of 10–25 μm decreased (from 35 to 30 %). Similar changes are observed in the sample from Buków 2—an increase in the proportion of 10–25 μm pores (from 52 to 54 %) and 1–10 μm pores (from 20 to 22 %), but a decrease in the proportion of 25–45 μm pores (from 17 to 13 %).

#### Sealing rocks

Petrophysical analyses showed that the sealing rocks (claystone from Zaosie 2 depth 912.40 m and mudstone from Buków 2 depth 1,436.00 m) are characterised by a higher specific surface area than the sandstones (in the range of 3.95–5.03 m^2^/g) and by low porosity of 3–5 % (Table 2). Rocks from the Zaosie 2 borehole show higher porosity values than the rocks from Buków 2. These rocks have similar values of threshold diameter at 0.05 μm and framework density of 2.54 to 2.57 g/cm^3^. The bulk density is 2.44 to 2.45 g/cm^3^. Predominant pore diameters in claystones from the Zaosie 2 and mudstone from Buków are below 0.1 μm (99 and 92 %, respectively) (Fig. 4).

Both of the sealing rock types (claystone and mudstone) experienced an impact on their petrophysical parameters as a result of the experiment. The porosity of each rock increased from 5 to 7.6 % in Zaosie 2, and from 3.1 to 9.1 % in Buków 2 (Table 2). The framework density slightly increased from 2.56 to 2.62 g/cm^3^ in Zaosie 2, and from 2.53 to 2.59 g/cm^3^ in Buków 2. The average capillary and threshold diameter also increased. The pore distribution in the pore space changed in favour of larger pores in each of the sealing rocks. In the sample from Zaosie 2, there was an increase in the percentage of 0.01–0.1 μm pores (from 76 to 82 %) and 1–0.1 μm pores (from 0.5 to 6 %) while the percentage of pores less than 0.01 μm significantly decreased (from 23 to 9 %) (Fig. 4). Changes in this parameter are also clearly apparent in the samples from the Buków 2 borehole and are manifested by the increase in the proportion of pores with diameters of 10–25 μm (from 1 to 6 %), 1–10 μm (from 1 to 11 %), and 0.1–1 μm (from 5 to 25 %) accompanied by a significant decrease in the proportion of pores less than 0.01 μm (from 45 to 14 %).

### Analyses of brines

The analyses of brines (Table 3) show an increase in TDS of solutions resulting from the experiments with both, sealing rocks and reservoir rocks after the experiment. This most probably resulted from dissolution of the rock matrix and the cements. The measured TDS is lower than the sum of ion concentrations due to partial outgassing of carbonates during the measurement. The significant increase in the concentration of bicarbonates results from dissolution of CO_2_ gas introduced during the experiment. Dissolution of the rock components also resulted in a slight increase in the concentrations of sulphates and calcium. In the case of the sealing rock, the significant increase in Ca^2+^ concentration after the experiment may indicate the presence of carbonate cements in the rock matrix.

**Table 3:** Chemical composition of brine solutions before and after the experiments

	Unit	Content in the brine sample before experiment	Content in the brine with sandstones after experiment	Content in the brine with sealing rocks after experiment
TDS	mg/dm^3^	2,420	3,578	3,094
Chlorides	mg/dm^3^ Cl^−^	741	755	798
Sulphates	mg/dm^3^ SO_4_^2−^	396	477	503
Bicarbonates	mg/dm^3^ HCO_3_^−^	195	1,904	1,269
Carbonates	mg/dm^3^ CO_3_^2−^	334	0.0	0.0
Calcium	mg/dm^3^ Ca^2+^	1.2	321	73.7
Magnesium	mg/dm^3^ Na^+^	807	910	919
Potassium	mg/dm^3^ K^+^	110	117	110

## Discussion

The 20-month long experiment on brine-rock-CO_2_ interaction, which was intended to determine the effect of injected CO_2_ on the petrophysical parameters of reservoir rocks and sealing rocks, showed variable changes in these parameters. In case of reservoir sandstones, the changes are smaller than for sealing rocks. Similar results were obtained in previous work by Fischer et al. (2010) and Tarkowski and Wdowin (2011).

The sandstones are characterised by a diversity of macropores. Pores larger than 1 μm make up more than 89 % of the pores. The threshold diameter value, which defines the pore size at which a continuous flow of media through the rock starts, is high in the sandstones—about 30 μm—indicating that these rocks show good hydraulic properties. These parameters as well as the framework density (2.6 g/cm^3^), bulk density (~2 g/cm^3^), and specific surface area (approximately 0.3 m^2^/g) did not change significantly during the experiment. A small decrease was observed only in the porosity. Similar results were obtained by Zemke et al. (2010) in a 15-month experiment on the impact of CO_2_ on rocks in the presence of brine. In the case of the sandstone from Zaosie 2 (838.10 m), the porosity decreased by only 0.5 %, while in the case of the sandstone from Buków 2 (1,435.5 m), the decrease was less than 4 %. Such phenomena are caused by precipitation of halite in pore spaces during drying of the rock samples. Similar results were obtained by Kaszuba et al. (2003).

The sealing rock series shows a variable structure. Some of the rocks exhibit compact and massive texture and low porosities of 3–5 %. They also contain a small percentage of pores greater than 1 μm (up to 3 %) and low threshold diameter values of 0.05 μm. In the sealing rocks, the changes caused by the experiment are more apparent than in the case of the sandstones. In both sealing rock samples, an increase in the porosity, the framework density, as well as the average capillary and threshold diameter was observed. The alterations are particularly pronounced in the sample of mudstone from the Buków 2 borehole from the depth of 1,436 m where the percentage of pores larger than 0.1 μm increased significantly from 52 to 86 %.

SEM-EDS observations of both reservoir rocks and sealing rocks showed no significant changes in a decrease in porosity as it was observed from petrophysical results. Feldspar skeletons indicating dissolution or kaolitinised feldspars indicating alterations were observed in the sandstones. It remains unclear which of the features observed on SEM images were formed as a result of primary alterations and which appeared due to the reactions during the experiment.

The results of brine analyses after the experiment for both the reservoir rock and sealing rocks indicate that the rock-brine-CO_2_ interaction resulted in an increase in concentration of ions indicating dissolution of the rock matrix and the cements. In the case of reservoir rocks, this is not a worrying phenomenon, but in sealing rocks this effect should be examined in more detail.

## Conclusions

The changes observed in the reservoir sandstones do not adversely affect the CO_2_ storage capacity. A slight decrease in the porosity (observed from petrophysical results) is due to precipitation of mineral phases (mainly halite in the pore spaces) that was precipitated from brine (Kaszuba et al. 2003). The lack of significant changes is mainly due to the lack of reactive minerals able to be corroded in the grain framework and due to the mild experimental conditions (low pressure of 6 MPa and temperature of 25 °C). To observe the changes in such experiments, it is desirable that the temperature is in the range of 150–200 °C. If the temperature is too low, i.e. 50–80 °C or lower, the changes may not be noticeable (Liu et al. 2012). In the case of claystones and mudstones (sealing rocks), the results showed that the experiment slightly affected the sample from the Zaosie 2 borehole, which did not worsen its sealing properties. However, the sealing properties of the sample from the Buków 2 borehole worsened significantly due to a considerable increase in the porosity and the percentage of pores above 1 μm in diameter. The reasons for these changes should be more thoroughly investigated.

These studies constitute only some of the steps necessary to determine the effects of interactions between rock, brine, and CO_2_ in order to evaluate the suitability of the rocks for carbon dioxide storage. The studies should be complemented by mineralogical and textural analyses as well as geochemical modelling to identify the changes caused by the impact of CO_2_ on the brine and rock matrix. This will allow for the ultimate determination of the suitability of the rocks from specified geological structures for CO_2_ sequestration.
